# The Association Between Infertility Treatment and Birth Outcomes for Nulliparous Persons Who Gave Birth 35 Years and Older: Findings from 2022 National Vital Statistics System Natality Data

**DOI:** 10.1007/s10995-025-04174-8

**Published:** 2025-09-01

**Authors:** Shanti U. Gallivan, Lynn M. Yee, Alexa Freedman, Joe Feinglass

**Affiliations:** 1 Program in Public Health, Northwestern University Feinberg School of Medicine, Chicago, USA; 2 Department of Obstetrics and Gynecology, Northwestern University Feinberg School of Medicine, Chicago, USA; 3 Department of Preventive Medicine, Northwestern University Feinberg School of Medicine, Chicago, USA; 4 Department of Medicine, Northwestern University Feinberg School of Medicine, Chicago, USA

**Keywords:** Infertility treatment, Advanced maternal age, Birth outcomes, Pregnancy outcomes, Health disparities

## Abstract

**Objectives:**

This study uses 2022 National Vital Statistics System natality data to identify characteristics associated with infertility treatment among nulliparous individuals 35 years or older, comparing pregnancy and birth outcomes between no infertility treatment and assisted reproductive technology (ART) or fertility-enhancing drugs or intrauterine insemination (IUI).

**Methods:**

The likelihood of infertility treatment was estimated after controlling for maternal age, education, race and ethnicity, insurance status, Women, Infants and Children (WIC) support, pre-pregnancy body mass index (BMI), chronic hypertension, diabetes, and smoking during pregnancy. Maternal outcomes (gestational diabetes, hypertensive disorders of pregnancy, cesarean birth, maternal morbidity) and neonatal outcomes (preterm birth, low birth weight, neonatal intensive care, and congenital anomalies) were compared for singleton and multifetal births separately.

**Results:**

Among 173,399 births, 13.6% had infertility treatment (10.9% ART, 2.4% IUI). As compared to people who identified as white or Asian, infertility treatment was over one-third less likely for non-Hispanic Black and Hispanic individuals and 2.4 times more likely for those with a graduate degree as compared to those with less than high school. Infertility treatment was associated with significantly higher rates of all adverse maternal and neonatal outcomes, and among multifetal births, ART was associated with a higher rate of maternal morbidity and more frequent gestational diabetes.

**Conclusions for Practice:**

Disparities in infertility treatment exist. ART was associated with modest but significantly worse outcomes, particularly for singleton births. Continued monitoring of infertility treatment selection and birth outcomes is needed for informed clinical and public policy decisions.

## Significance

What is already known about the subject?

What this study adds?

Older nulliparous individuals are a birthing population with high rates of infertility treatment. With expanded availability of fertility treatment and advances in clinical techniques, it is important to understand population-based infertility treatment outcomes.

This study of 2022 births provides a sociodemographic and clinical profile of infertility treatment use, comparing outcomes for singleton and multiple births. Fertility treatment was associated with modest but significantly worse maternal and fetal outcomes. Findings highlight ongoing disparities in access to fertility treatment and estimates of the magnitude of risk associated with each type of treatment.

## Introduction

Age at first birth in the United States (US) has risen from 24.2 years in 1990 to 27.3 years in 2021 (Mathews & Hamilton, 2002; Osterman et al., 2023). In 2021, 19.9% of all births occurred among those age 35 or older compared to 14.5% in 2010 (Martin et al., 2012; Osterman et al., 2023). This shift in maternal age reflects delayed childbearing due to advances in women’s education and economic status, assisted by the increased use of infertility treatments such as assisted reproductive technology (ART) and fertility-enhancing drugs or intrauterine insemination (IUI). In 2018, 203,119 ART procedures were performed, resulting in 73,831 live-birth deliveries and 81,478 infants born (Sunderam et al., 2022). Infertility treatment has also improved, with lower rates of multifetal births. In 2006, 52% of ART births were singletons, compared to 78.7% in 2018 (Sunderam et al., 2009, 2022).

As the use of infertility treatment increases, it is important to understand and monitor recent birth outcomes associated with this therapy, especially for high-risk groups such as nulliparous individuals age 35 or older who compose almost two-thirds of those who use ART (Jackson et al., 2015; Johnston et al., 2015; Prevention, 2024; Tierney & Cai, 2019). Older studies of the association of ART with birth outcomes have found that people who conceived using ART were at higher risk of adverse neonatal outcomes such as preterm birth, low birth weight, congenital anomalies, and perinatal mortality (Hansen et al., 2012; Helmerhorst et al., 2004). More recent studies, including a 2020 metanalysis, have also found that ART singleton births across all maternal age and parity groups had an increased risk of adverse birth outcomes compared to the general population (Tierney & Cai, 2019; Wennerholm & Bergh, 2020). Although nulliparous individuals represent less than one-third of all births to individuals age 35 or older, these individuals are at higher risk for adverse outcomes and have different pregnancy experiences (Aasheim et al., 2014; Bayrampour et al., 2012; Osterman et al., 2023). However, few studies provide separate analyses of singleton versus multifetal birth outcomes, particularly in an older, nulliparous population.

This study analyzes 2022 National Vital Statistics System (NVSS) natality data to study use of infertility treatment for older individuals experiencing their first birth. Our first aim was to describe sociodemographic and clinical characteristics of nulliparous birthing individuals 35 years and older who used infertility treatment in 2022, including both ART and IUI, compared with those who did not use infertility treatment. Our second aim was to compare a range of maternal and neonatal pregnancy and birth outcomes by infertility treatment type, controlling for maternal sociodemographic and clinical characteristics, and separating outcomes by singleton versus multifetal pregnancies. Findings provide evidence about potential improvements in disparities and outcomes as access and treatment approaches have evolved in recent years.

## Methods

Using the National Vital Statistics System (NVSS) natality file for US births in 2022, we identified births of nulliparous people aged 35 or older with live births. NVSS is a cooperative effort between the National Center for Health Statistics and states (Statistics, 2024). In this analysis, birth records with missing information on maternal age, infant birth weight, parity, use of infertility treatment, or maternal education were excluded.

The NVSS separately categorizes infertility treatment as either in vitro fertilization (i.e., ART) or fertility-enhancing drugs with or without intrauterine insemination (hereafter referred to as IUI). Sociodemographic variables include maternal age (35–39, 40–45, and ≥ 45 years). Maternal race and ethnicity, understood to be social constructs, were grouped as Non-Hispanic white, Non-Hispanic Black, Hispanic, Asian, and other, which included birthing individuals of multiple races, American Indian and Alaska Native, and Native Hawaiian or Other Pacific Islander. Maternal education was grouped as less than high school, completed high school, some college, completed a four-year university or college degree program, or completed a graduate degree. NVSS natality data also includes whether the Special Supplemental Nutrition Program for Women, Infants, and Children (WIC) program was used during the pregnancy and if the payment source for the delivery was Medicaid versus private or other insurance, both markers for socioeconomic status. Smoking during pregnancy was defined as reported smoking any cigarettes during their first, second, or third trimester. Because marital status was missing for California data (13% of births), we did not include it in our analyses.

Clinical characteristics included pre-pregnancy diabetes, chronic hypertension, and pre-pregnancy body mass index (BMI) categorized as underweight (< 18.5), normal weight (18.5–24.9), overweight (25–29.9), and obese (≥ 30). For analyses of multifetal births, we compared twins to triplets and higher order births.

Maternal outcomes included gestational diabetes, hypertensive disorders of pregnancy (HDP; defined as gestational hypertension or preeclampsia/eclampsia), cesarean birth, and maternal morbidity. Maternal morbidity in the natality file includes transfusion, perineal laceration, ruptured uterus, unplanned hysterectomy, and if admitted to intensive care. Neonatal outcomes included preterm (< 37 weeks) and very preterm (< 32 weeks) birth, low birth weight (< 2500 g) and very low birth weight (< 1500 g), admission to neonatal intensive care unit (NICU), or any congenital anomalies. Congenital anomalies in the natality file include anencephaly, meningomyelocele, cyanotic congenital heart disease, hernia, omphalocele, gastroschisis, limb, cleft lip (with or without palate), cleft palate alone, Down syndrome, suspected chromosomal disorder, and hypospadias.

The bivariate significance of differences in maternal characteristics for use of ART or IUI was analyzed with chi square tests. Multivariable logistic regression was used to test the likelihood of any infertility treatment controlling for all maternal sociodemographic and clinical characteristics. Bivariate analyses of the association of birth outcomes with infertility treatment type were stratified by singleton vs. multifetal gestation due to the well-known greater risk of adverse outcomes among multifetal gestation. Chi square tests were used for bivariate comparisons of birth outcomes by fertility treatment type versus no treatment. Poisson regression, adjusted for maternal age, race and ethnicity, marital status, WIC support, Medicaid coverage of the delivery, pre-pregnancy BMI, chronic hypertension, diabetes and smoking during pregnancy and (for multifetal births) triplet or higher order births, was used to estimate incidence rate ratios (IRRs) for all 10 pregnancy or birth outcomes. The IRR more closely approximates relative risk than odds ratios given the incidence of birth outcomes analyzed (Katz, 2006; Zou, 2004). Because multifetal births are not linked to maternal identifiers in the natality file, we compared multiple gestation birth outcomes by infertility treatment without accounting for clustering, treating each birth record as an independent observation. Coefficients were obtained for each type of infertility treatment (with no infertility treatment as the reference).

## Results

### Sociodemographic and Clinical Differences in the Likelihood of Infertility Treatment

**Fig. 1:**
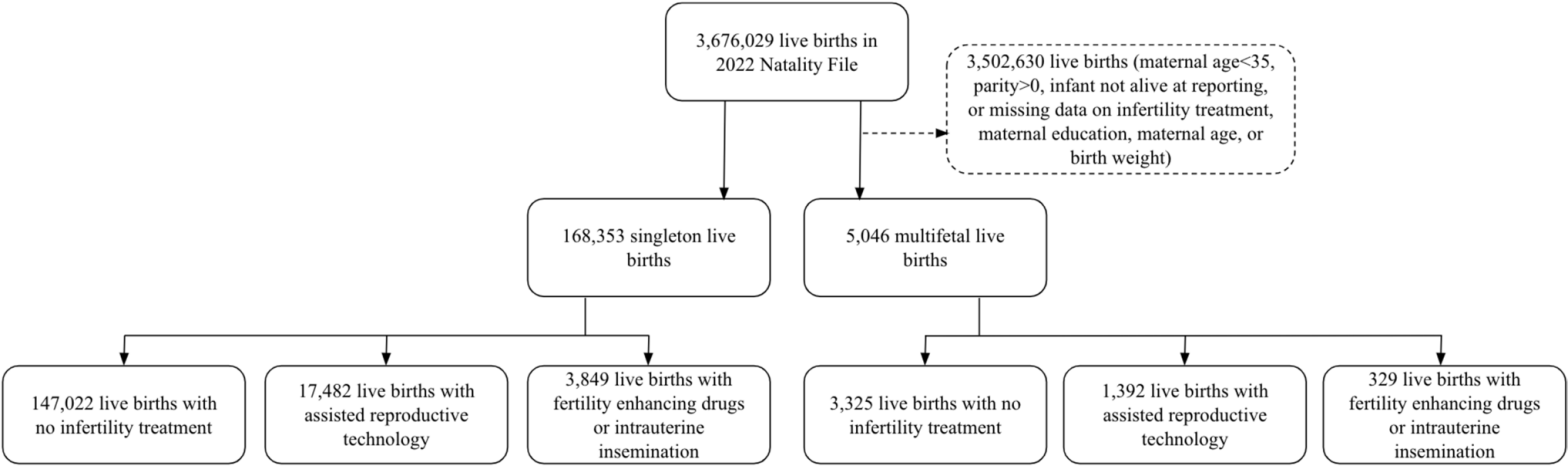
Study inclusion and analysis groups

After excluding 2.2% of birth records for missing information on infant birth weight (0.1%), parity (0.2%), utilization of infertility treatment (0.2%), or maternal education (1.7%) there were 173,399 2022 births to the nulliparous age 35 + population (Fig. 1). There were 23,584 (13.6%) births with any infertility treatment (Table 1). Prevalence of infertility treatment was 10.9% for ART and 2.4% for IUI. The use of infertility treatment was 11.1% for age 35–39 individuals rising to 22% of those age 40–44 and 51.7% of those age ≥ 45. The use ART compared to IUI was higher for all age groups, with the highest difference occurring for individuals age ≥ 45 (45.8% using ART and only 5.0% using IUI). The proportion of multifetal births was 7.5% for any infertility treatment,7.4% for ART and 7.9% for IUI versus 2.2% for no infertility treatment births.

**Table 1:** Utilization of infertility treatment for births among nulliparous persons 35 years and older by sociodemographic and clinical characteristics*

	Column percent all births N = 173,399	Row percent any infertility treatment N = 23,584	Row percent assisted reproductive technology N = 18,874	Row percent fertility drugs/intrauterine insemination only N = 4,178	Odds ratio for any infertility treatment (95% confidence interval)
All Births	100.0	13.6	10.9	2.4	
Maternal Age					
35–39	81.9	11.1	8.6	2.2	Reference
40–44	16.4	22.3	18.9	3.0	2.42 (2.34–2.51)
45 +	1.7	51.7	45.8	5.0	9.44 (8.75–10.20)
Race and ethnicity					
Non-Hispanic White	56.9	15.0	11.8	2.9	Reference
Non-Hispanic Black	10.3	9.6	7.7	1.7	0.62 (0.59–0.66)
Hispanic	16.9	8.9	6.9	1.8	0.66 (0.63–0.69)
Asian	13.5	16.8	14.5	2.1	1.10 (1.06–1.15)
None of the above	2.5	12.2	10.2	1.9	0.82 (0.75–0.91)
Maternal education					
Less than high school	2.4	4.5	3.4	1.0	Reference
High school	8.5	6.9	5.3	1.4	1.20 (1.01–1.41)
Some College	18.2	9.2	7.0	1.9	1.35 (1.16–1.58)
Bachelor’s	35.4	13.9	11.2	2.4	1.84 (1.58–2.15)
Graduate Degree	35.5	17.8	14.4	3.0	2.35 (2.02–2.75)
Received WIC Support	10.1	5.0	3.7	1.1	0.67 (0.62–0.72)
Medicaid Delivery	13.5	4.5	3.5	1.0	0.44 (0.41–0.47)
Pre-pregnancy BMI					
Underweight	1.9	12.3	10.3	1.7	0.75 (0.67–0.84)
Normal weight	42.5	14.1	11.6	2.2	0.86 (0.83–0.90)
Overweight	27.1	13.3	10.7	2.3	0.88 (0.85–0.92)
Obese	26.9	13.5	10.2	2.9	Reference
Pre-pregnancy diabetes	1.8	13.7	10.2	2.9	1.08 (0.96–1.20)
Chronic hypertension	4.8	16.4	13.0	3.2	1.29 (1.21–1.37)
Smoking during pregnancy	1.8	1.8	1.0	0.7	0.18 (0.14–0.24)

National Vital Statistics System 2022 Natality File

*All bivariate comparisons of any infertility and no fertility treatment p < 0.001 except pre-pregnancy diabetes

The highest infertility treatment rates were for Asian and white individuals, with non-Hispanic Black and Hispanic individuals having over a 38% lower likelihood. Infertility treatment was highly correlated with maternal education. After adjustment for covariates, infertility treatment was 2.4 times more likely for individuals with a graduate degree versus those with less than high school. Those with lower socioeconomic status (i.e. WIC or Medicaid-covered deliveries) or who smoked during pregnancy were much less likely to have had infertility treatment, whereas those with pre-pregnancy obesity and chronic hypertension were more likely.

### Differences in Pregnancy and Birth Outcomes

Table 2 presents bivariate comparisons for the 10 maternal and neonatal outcomes by infertility treatment type, presented separately for singleton and multifetal births. Singleton birth outcomes were consistently better for no infertility treatment births (all treatment type comparisons p < 0.001 except for very low birth weight). The largest differences in singleton outcomes between ART and IUI were for the likelihood of a cesarean birth, preterm birth, low birth weight and NICU admission.

**Table 2:** Birth outcomes by use of infertility treatment for singleton and multiple gestation births

	Column percent all births	Row percent no infertility treatment	Row percent assisted reproductive technology	Row percent fertility drug/intrauterine insemination only
Singleton births	N = 168,353	N = 147,022	N = 17,482	N = 3,849
Cesarean Birth**	46.8	45.3	58.1	51.4
Gestational Diabetes**	11.7	11.4	14.2	14.0
Any Hypertensive Disorders of Pregnancy**	13.6	13.2	16.5	16.1
Any Maternal Morbidity^1^**	2.4	2.2	3.7	3.6
Preterm Birth (< 37 weeks)**	12.6	12.2	15.6	13.3
Very or Extremely Preterm Birth (< 32 weeks)**	2.0	1.9	2.4	2.1
Low Birth Weight (< 2500 g)**	9.6	9.5	10.5	9.1
Very Low Birth Weight (< 1500 g)	1.6	1.6	1.8	1.7
Admission to Neonatal Intensive Care Unit**	11.8	11.4	14.8	12.3
Any Congenital Anomolies^2^**	0.4	0.3	0.5	0.5

Multifetal births	N = 5,046	N = 3,325	N = 1,392	N = 329
Triplet or Higher Order Birth*	2.6	2.4	3.5	0.9
Cesarean Birth*	86.6	85.7	88.9	85.7
Gestational Diabetes*	14.5	13.2	17.0	17.6
Hypertensive Disorders of Pregnancy*	23.8	22.5	26.4	25.5
Any Maternal Morbidity*	2.8	2.1	4.5	2.4
Preterm Birth (< 37 weeks)	60.2	60.2	59.5	62.0
Very or Extremely Preterm Birth (< 32 weeks)	11.5	11.5	11.8	10.3
Low Birth Weight (< 2500 g)	60.1	60.4	61.0	55.0
Very Low Birth Weight (< 1500 g)	10.1	10.1	10.8	7.9
Admission to Neonatal Intensive Care Unit	46.1	45.4	47.5	47.1
Any Congenital Anomalies	0.3	0.3	0.1	0.6

National Vital Statistics System 2022 Natality File

**Bivariate comparisons p < 0.001 for singleton births except for very low birth weight

*Bivariate comparisons p < 0.01 for multifetal births

^1^Maternal Morbidity includes maternal transfusion, perineal laceration, ruptured uterus, unplanned hysterectomy, and admitted to intensive care

^2^Any congenital birth defects include anencephaly, meningomyelocele, cyanotic congenital heart disease, hernia, omphalocele, gastroschisis, limb, cleft lip (with or without palate), cleft palate alone, down syndrome, suspected chromosomal disorder, and hypospadias

Among multifetal gestations, triplets or higher-order births were 1.4% higher for ART as compared to no treatment, but lower among the small group (6.5%) of IUI births. Gestational diabetes was higher for multifetal births with infertility treatment. As compared to no treatment, maternal morbidity was over twice as high for ART but similar for IUI. Cesarean birth, triplet or higher order births, and HDP comparisons were p < 0.01 but prematurity, low birth weight, NICU admission and birth defects rates were not statistically significant for multiple gestation births.

### Association Between Infertility Treatment Type and Pregnancy and Birth Outcomes

Table 3 presents regression results for the likelihood of birth outcomes for both singleton and multifetal births, showing results for each type of infertility treatment as compared to no infertility treatment, adjusted for all sociodemographic and clinical characteristics. Among singleton births, those who conceived using either type of infertility treatment were more likely to have a cesarean birth, to develop gestational diabetes or HDP, and to have maternal morbidity compared with those who did not use infertility treatment. Both infertility treatment types were also associated with greater likelihood of preterm birth and NICU admission, although differences between no infertility treatment and IUI were more modest.

**Table 3:** Poisson regression results^3^ for birth outcomes for singleton and multiple gestation births infertility treatments compared to no infertility treatments

	Assisted reproductive technology incidence rate ratio (95% CI)	Fertility drugs/intrauterine insemination only incidence rate ratio (95% CI)
Singleton births		
Cesarean Birth	1.24 (1.21–1.27)	1.11 (1.06–1.16)
Gestational Diabetes	1.22(1.17–1.28)	1.19(1.09–1.30)
Hypertensive Disorders of Pregnancy	1.28(1.22–1.33)	1.17(1.08–1.26)
Any Maternal Morbidity	1.61 (1.48–1.76)	1.61 (1.36–1.91)
Preterm Birth (< 37 weeks)	1.32 (1.27–1.38)	1.12 (1.02–1.22)
Very or Extremely Preterm Birth (< 32 weeks)	1.44 (1.29–1.60)	1.21 (0.96–1.51)
Low Birth Weight (< 2500 g)	1.14 (1.08–1.20)	1.00 (0.90–1.11)
Very Low Birth Weight (< 1500 g)	1.33 (1.17–1.50)	1.18 (0.92–1.51)
Admission to Neonatal Intensive Care Unit	1.36 (1.30–1.42)	1.10 (1.01–1.21)
Any Congenital Anomalies	1.46 (1.15–1.85)	1.34 (0.84–2.15)
Multifetal births		
Triplet or Higher Order Birth	1.45 (1.00–2.11)	0.46 (0.14–1.45)
Cesarean Birth	1.03 (0.96–1.10)	1.01 (0.89–1.14)
Gestational Diabetes	1.21 (1.03–1.42)	1.36 (1.03–1.79)
Hypertensive Disorders of Pregnancy	1.21 (1.06–1.37)	1.09 (0.87–1.37)
Any Maternal Morbidity	2.08 (1.46–2.96)	1.06 (0.51–2.21)
Preterm Birth (< 37 weeks)	0.97 (0.90–1.06)	1.03 (0.89–1.19)
Very or Extremely Preterm Birth (< 32 weeks)	1.10 (0.91–1.33)	0.98 (0.69–1.39)
Low Birth Weight (< 2500 g)	1.01 (0.93–1.09)	0.94(0.80–1.09)
Very Low Birth Weight (< 1500 g)	1.18 (0.97–1.44)	0.90 (0.60–1.34)
Admission to Neonatal Intensive Care Unit	1.03 (0.94–1.13)	1.03 (0.88–1.22)
Any Congenital Anomalies	0.58 (0.12–2.68)	2.32 (0.50–10.81)

National Vital Statistics System Natality 2022 File

^3^Poisson regression model adjusted for age, maternal education, race and ethnicity, WIC support, Medicaid coverage for delivery, pre-pregnancy BMI, pre-pregnancy diabetes, chronic hypertension, and smoking during pregnancy

Among multifetal births, both types of infertility treatment were associated with greater risk of gestational diabetes. HDP and maternal morbidity were also more likely for those using ART but not for those using fertility-enhancing drugs/IUI. Risk of preterm birth, low birth weight, cesarean birth, and NICU admission were not statistically significantly associated with either type of infertility treatment among those with multifetal births. Among the subgroup of those with triplet or higher birth orders, gestational diabetes, HDP and maternal morbidity rates were significantly higher for people with multifetal births who conceived using ART although much less elevated for IUI (data not shown).

## Discussion

In 2022, nulliparous birthing individuals age 35 years and older who had (successful) infertility treatment were predominantly white or Asian and more highly educated. The social class gradient in use of infertility treatment was reflected in much lower rates for those with WIC support and Medicaid covered deliveries. These findings provide a benchmark for future efforts to reduce infertility treatment barriers.

We also found that singleton births conceived using ART had modestly higher rates of adverse birth outcomes, particularly cesarean birth, congenital anomalies, and very low birth weight and maternal morbidity. In contrast to no infertility treatment, most singleton birth adverse events were more likely for ART than for IUI, especially preterm and low birth weight births. Multifetal births conceived using ART were associated with higher rates of gestational diabetes and maternal morbidity, however multiple gestation ART congenital anomalies were lower than for IUI births. IUI births had generally lower rates of adverse outcomes than ART, but higher adverse outcome rates than births with no infertility treatment. While only 13.6% of the births in our sample were associated with infertility treatment, this included 34% of the multiple gestation births, including a one-third higher rate of triplet or higher order births.

### Fertility Treatment Disparities

The results of our study are consistent with previous literature showing a higher likelihood of white and Asian, more highly-educated individuals to use infertility treatment, reflecting persistent challenges of equitable access to reproductive health services in the US (Dongarwar et al., 2022; Kessler et al., 2013). A 2011–2019 NVSS natality study found that, as compared to white individuals, non-Hispanic Black and Hispanic individuals both had an age-adjusted 70% lower likelihood of utilizing infertility treatment as compared with white individuals (Dongarwar et al., 2022). This compares to a 31% and 35% lower likelihood in our 2022 study of the older nulliparous population. These smaller gaps in infertility treatment births potentially suggest increased access to infertility treatment. Advances in access are consistent with increased mandated insurance coverage for infertility treatments and sociodemographic differences in this population compared to the general population (Bitler & Schmidt, 2012; Imrie et al., 2023; Peipert et al., 2022).

Among those with an infertility diagnosis, high desire for parenthood, agreement from partner, accessibility to emotional and social support and higher health literacy have been identified as the most important factors driving treatment seeking (Cebert-Gaitors et al., 2022; Olerich et al., 2021). These considerations may also drive some of the observed disparities in treatment. However, cost is the most reported barrier to care across race/ethnicity and insurance status (Insogna et al., 2020; Olerich et al., 2021). Many birthing individuals do not know if their insurance covers infertility treatment, especially Hispanic/Latino individuals (Insogna et al., 2020). Other research has identified a lack of access due to work schedules or the travel distance to infertility clinics (Olerich et al., 2021). Other common barriers to seeking infertility treatment include internalized stigma, limitations of professional careers, a lack of social support for the treatment decision, and negative perceptions of the clinical environment (Cebert-Gaitors et al., 2022). Although our study does not evaluate patient-reported factors, our data corroborate the persistent presence of disparities by race and class that are heavily associated with health care access and health outcomes across all domains (Imrie et al., 2023).

Our findings show the critical importance fertility treatment to allowing individuals to become parents. Older nulliparous birthing individuals have both lower infertility treatment success rates and higher risk for adverse birth outcomes (Braggion et al., 2023; Elci et al., 2022; Ubaldi et al., 2019). Patients who are over 35 years of age make up the majority of those who use ART. In 2021, of those who used ART, 36.2% were < 35, 23.4% were 35–37, 20.7% were 38–40, and 19.7% were > 40 years old (Office, 2024). The success of ART decreases by age. In 2021, the percentage of ART retrievals that resulted in deliveries for individuals under ager 35 age was 53.7% compared to 39.7%, 26.4%, and 9.4% for 35–37, 38–40, and over 40 years old, respectively (Team, 2024). As population-wide fertility declines, it is increasingly important to understand birthing patterns among 35+ individuals as they become a larger birthing population, especially with the expanding use of infertility treatment (Adashi et al., 2024).

### Fertility Treatment Outcomes

Adverse birth outcomes after fertility treatment are thought to be related to multiple etiologies, for instance, conditions of the uterus and ovaries, as well as to complications of ART procedures (Lei et al., 2019; Yu et al., 2022). We indeed found that infertility treatment was higher among those with pre-pregnancy obesity, chronic hypertension, and pre-pregnancy diabetes (but significantly lower among those who smoked during pregnancy, likely reflecting planned, wanted pregnancy) (He & Wan, 2023). It is thus not possible to directly infer birth outcome causality from treatment.

Adverse outcomes observed in this study are consistent with previous literature, summarized in a 2016 systematic review of evidence on the association of IVF treatment with adverse pregnancy and perinatal outcomes (Palomba et al., 2016). Previous studies have documented higher rates of cesarean birth, gestational diabetes, preterm birth, low birth weight, and maternal morbidity associated with infertility treatment for singleton births, as well as severe maternal morbidity related to a higher number of multiple births (Luke, 2017; Passet-Wittig & Greil, 2021; Richmond et al., 2022). Canadian population-based studies from over a decade ago found similarly higher cesarean birth rates, higher maternal morbidity, and higher NICU admissions associated with infertility treatment (Dayan et al., 2019; Richmond et al., 2022). A 2022 retrospective cohort study looking at all neonates born in Utah from 2009 to 2017 found that neonates conceived through medically assisted reproduction weighed less, were born earlier, and were more likely to be LBW, preterm, and small for gestational age (Pelikh et al., 2022). However, researchers noted that these differences were unlikely to be directly caused by infertility treatments but were instead related to underlying fertility problems and health conditions (Pelikh et al., 2022).

Of course, the higher likelihood of multifetal pregnancies after infertility treatment, in itself, conveys increased risk for maternal and neonatal complications. When focusing on comparisons of just multifetal birth outcomes, Canadian population-based studies from over a decade ago also found similarly higher cesarean birth rates, higher maternal morbidity, and higher NICU admissions associated with multifetal birth after infertility treatment (Dayan et al., 2019; Richmond et al., 2022). Despite recent declines in population-wide triplet and higher-order birth rates due to advances in infertility treatment, there was over a 5% difference in multifetal births, and amongst multifetal births, there was a 46% higher rate of triplets or higher order births for ART (Anderer, 2024).

### Strengths & Limitations

Strengths of this analysis include its large, representative sample with contemporary data. However, there are important limitations. The accuracy of ART classification is limited due to the NVSS use of birth certificate data. Previous studies have found birth certificate data to be an unreliable source for identifying infertility treatment type and have documented underreporting of infertility treatment usage (Luke et al., 2016; Lynch et al., 2011). However, NVSS data provides additional detail on birth outcomes and maternal clinical characteristics often missed in other infertility treatment sources and does not rely on self-reporting (Ahluwalia et al., 2013; Dietz et al., 2014). These data, based on live birth certificate information, do not address potentially important differences in fetal death. Differences in birth outcomes reflect the fact that those who received infertility treatment had worse pre-pregnancy health status than those with no treatment. We cannot infer how much treatment itself contributes to adverse outcomes. Second, our results about access to treatment reflect only successful infertility treatment that results in a live birth. We do not know about those who used infertility treatment but did not have a live birth, or the extent to which infertility treatment success is itself linked to the same sociodemographic and clinical characteristics associated with treatment. Not all birthing people who use infertility treatment are infertile. LGBTQ+ families utilize these services frequency to achieve their family planning goals. (Kirubarajan et al., 2021). A true estimate of the magnitude of population-based disparities in infertility treatment there would require data on the total population with infertility who desire conception and the proportion of those individuals across various population groups. This information is not available in the US. Third, because multifetal births are not linked to maternal identifiers in the natality file, we had to compare multifetal birth outcomes by infertility treatment without clustering, treating each multifetal birth as a double-counted observation. By separately analyzing singleton and multifetal births, we reduce the estimates of overall risk of adverse birth outcomes related to a higher likelihood of multifetal births after infertility treatment. Finally, birth outcomes such as maternal morbidity do not approximate more commonly used measures such as severe maternal morbidity and represent a diverse spectrum with different etiologies which differ by type of infertility treatment.

## Conclusion

When compared to births with no infertility treatment, this study documented a modest but significant burden of adverse birth outcomes associated with successful infertility treatment, particularly ART. With delayed childbearing and advances in infertility treatment technology, birthing individuals age 35+ are a growing population. The additional risk that infertility treatment places on births from this population must continue to be part of counseling and shared decision making based on the best available evidence. As infertility treatment use continues to increase, assessing equity of access to treatment is an ongoing concern for both public policy and health system quality improvement and health equity initiatives. Of particular interest is the influence of the rise in obesity in the reproductive age population on both access to infertility treatment and deteriorating pregnancy outcomes (Wang et al., 2021). Finally, another important future research aim is to determine the extent of geographic variation in access to infertility treatment related to specific state legislative and insurance restrictions (Harris et al., 2017; Mackay et al., 2023), as part of a comprehensive research agenda to improve outcomes for patients from the most vulnerable reproductive age populations.

## Data Availability

The data supporting the findings of this study are publicly available from the National Vital Statistics System (NVSS), Center for Disease Control website.

## Code Availability

The data analysis was conducted using STATA Version 17 software. Analysis scripts (do-files) are available upon request.
